# Optimal PID Control of a Brushed DC Motor with an Embedded Low-Cost Magnetic Quadrature Encoder for Improved Step Overshoot and Undershoot Responses in a Mobile Robot Application

**DOI:** 10.3390/s22207817

**Published:** 2022-10-14

**Authors:** Ricard Bitriá, Jordi Palacín

**Affiliations:** Robotics Laboratory, Universitat de Lleida, Jaume II 69, 25001 Lleida, Spain

**Keywords:** PID control, brushed DC motor, magnetic encoder, omnidirectional mobile robot

## Abstract

The development of a proportional–integral–derivative (PID) control system is a simple, practical, highly effective method used to control the angular rotational velocity of electric motors. This paper describes the optimization of the PID control of a brushed DC motor (BDCM) with an embedded low-cost magnetic quadrature encoder. This paper demonstrates empirically that the feedback provided by low-cost magnetic encoders produces some inaccuracies and control artifacts that are not usually considered in simulations, proposing a practical optimization approach in order to improve the step overshoot and undershoot controller response. This optimization approach is responsible for the motion performances of a human-sized omnidirectional mobile robot using three motorized omnidirectional wheels.

## 1. Introduction

Currently, the mechanical power required to move or operate mobile robots is generated by direct current (DC) motors that convert the electrical power to mechanical power. Even hydraulically activated humanoid robots such as Atlas [1] use a DC current motor to drive a compact hydraulic pump that delivers high power to its 28 hydraulic joints. The basis of a DC motor relies on the forces produced by two magnetic fields, of which at least one of them is electrically generated. There are three basic types of DC motors [2,3]: brushed DC motors (BDCMs), steppers, and brushless DC motors (BLDCMs). BDCMs are characterized by the use of an internal switching mechanism based on brushes that automatically creates a rotating magnetic field that directly generates angular kinetic energy. BDCMs automatically start to rotate at the maximum speed when plugged-in as they are basically designed to operate efficiently and provide the maximum torque when rotating at a given nominal speed (around 3000 rpm). BDCMs are normally combined with a gearbox to obtain the angular rotational speed needed in the robotic application. The operational angular rotational speed can be reduced linearly by linearly reducing the nominal DC voltage applied to the BDCM. Steppers do not have any internal switching mechanism so they remain in a fixed static position when plugged-in, requiring external switching to rotate. Steppers are efficiently designed to provide the maximum torque at zero or very low angular rotational speeds and normally are not combined with a gearbox. The angular rotational speed of a stepper is fixed by the frequency of the switching sequence. Steppers do not usually have sensors in the rotor so they are usually accelerated and decelerated using very conservative profiles in order to give time to the motor to follow these profiles. BLDCMs do not have any internal switching mechanism and are characterized by the use of three internal inductors that are used jointly to create a rotating magnetic field. BLDCMs are efficiently designed to provide a high mechanical torque at zero, very-low, high, and very high (more than 3000 rpm) angular rotational velocities. BLDCMs can use a gearbox depending on the maximum angular rotational velocity required by a given application. A BLDCM used with DC power requires an external electronic switch tailored to optimally control the timing of the power applied to the three internal inductors. The efficient control of the acceleration and deceleration of a BLDCM requires the use of sensors in the rotor (normally hall detectors) in order to apply the optimal switching sequence and control the motor. Finally, in some application contexts a servo-motor is also considered as another DC motor type [4,5], although this compact device is internally a combination of a DC motor, a position or velocity sensor, and a control system in one single package.

This paper is focused on the control of the angular rotational velocity of a BDCM, which is a relatively inexpensive motor that is widely used in mobile robotic applications and developments [6,7]. The main disadvantage of BDCMs is that there is no direct method for angular rotational velocity regulation that depends on the power applied and the instantaneous torque required by the load. Thus, a BDCM must be complemented with a sensor able to measure its real angular rotational velocity and a control system able to compare the real and target angular velocities in order to regularly update the power applied to the motor and regulate its angular rotational velocity. In this regard, a proportional–integral–derivative (PID) controller continuously calculates the error or difference between the desired angular velocity setpoint and the estimation of the real angular velocity of the motor and then computes a correction based on the proportional, integral, and derivative evaluation of this difference.

The concept of the PID controller has been extensively used since its first formal definition by Minorsky [8] and has evolved from the early pneumatic versions to analog electronic controllers and the more recent digital implementations [9,10]. The optimal use of a PID controller requires the appropriate tuning of the weights of the internal proportional, integral, and derivative terms of the controller according to some optimization criteria, which is not a trivial problem [11,12,13]. One of the most known tuning methods is the Ziegler-Nichols’s method presented in 1942 [14], which provides fast and simple rule-based tuning [13,15], although there are many other design methods such as the internal model control [16,17] or gain margin, phase margin, and gain crossover frequency methods [18,19]. Garrido et al. [20] proposed a method for the iterative design of centralized PID controllers based on equivalent loop transfer functions and linear programing. Euzébio et al. [21] proposed a nonlinear optimization algorithm to minimize the disturbance effects in coupled loops in decentralized PID controllers. Torga et al. [22] proposed a nonlinear optimization for the simultaneous tuning of control loops in a cascade configuration under load disturbance.

The specific problem of reducing the PID step overshoot response has been addressed by many approaches [12], such as by analyzing the relation between the location of the transfer function poles and the overshoot [23], by using sliding perturbation observers [24], by implementing a Tabu search algorithm [25], or by explicitly avoiding the overshoots [26]. The versatility of the basic core algorithm of the PID controller has fostered many enhancements [27] and its application to control dynamic systems [28]. For example, Podlubny [29] proposed a PID implementation involving a fractional-order integrator and a fractional-order differentiator (PI^λ^D^μ^) in order to control systems that are better described by fractional-order mathematical models, a proposal that has been applied successfully to control a quadrotor unmanned aerial vehicle (UAV) [30] and to control the position of a micrometric linear axis [31].

**Figure 1:**
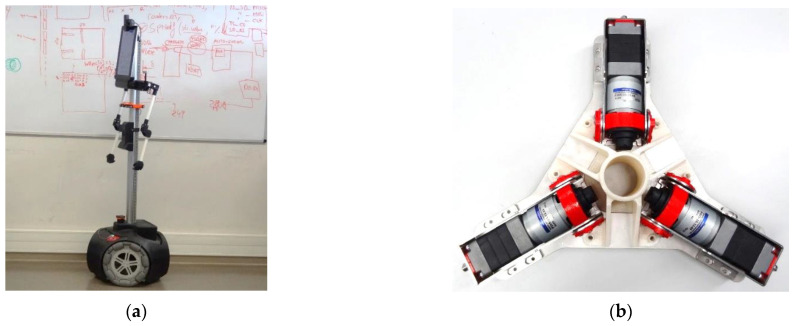
(**a**) Image of the APR-02 mobile robot. (**b**) Details of the internal structure supporting the three BDCMs that drive the three omnidirectional wheels of the mobile robot.

In certain applications such as mobile robots, most research papers usually state the use of PID controllers without completely describing the PID implementation details, the tuning procedure, or the encoder used (or planned) to measure the velocity of the motors [32,33,34,35,36,37]. The main contribution of this paper is the complete and accurate practical description of the PID controllers implemented in a medium-sized (1.76 cm tall) omnidirectional mobile robot, which uses three BDCM to directly drive its three omnidirectional wheels (Figure 1). As is well known, estimating of the position and trajectory of a mobile robot [38,39] heavily relies on the performance of the design of the wheels [40,41,42] and on the accurate control of the rotation of the wheels [43,44], because small differences can lead to noticeable errors in the final position reached by the mobile robot. The use of an omnidirectional motion system in a mobile robot allows superior maneuverability and the imitation of human mobility [45,46], but the control of the angular rotational velocity of the wheels must be very accurate in order to reduce odometry errors [47,48]. This paper describes the PID tuning procedure used in this mobile robot and all of the implementation details. The factors described and analyzed are the correction of the angular velocity information provided by a low-cost magnetic quadrature encoder, the modeling of the brushed DC motor (BDCM), the PID implementation, the anti-wind-up strategy used, the sampling time determination, the basic PID tuning procedure, and the optimization of the PID parameters for improved step overshoot and undershoot responses tailored for a mobile robot application. As described in this paper, the feedback provided by the magnetic encoders produces some control artifacts that reduce the effectivity of the PID control, so the usefulness of the proposed PID optimization is demonstrated through a practical experimental analysis. The optimal PID tuning procedure described in this paper defines the motion performances of the APR-02 human-sized, three-wheeled omnidirectional mobile robot [47,48].

## 2. Materials and Methods

The Materials and Methods used in this paper are the APR-02 mobile robot, the electronic board implementing the PID controller, the BDCM used in the mobile robot which includes a low-cost magnetic encoder, the PID control method, and the error function used to optimize the tuning of the PID procedure.

### 2.1. Omnidirectional Mobile Robot APR-02

Figure 1a shows an image of the APR-02 mobile robot prototype used in this paper as an application example. This robot was developed at the Robotics Laboratory in University of Lleida under the concept of an assistant personal robot (APR), firstly described by Clotet et al. [49]. This three-wheeled omnidirectional mobile robot concept (Figure 1b) was originally designed as a tool to evaluate teleoperation services, while its current APR-02 prototype version [50] is able to operate autonomously in unconstrained scenarios. The APR-02 mobile robot has already been applied as a tool for the automatic supervision of temperature, humidity, and luminance [51]; as a walk-helper tool [52]; and as a mobile robot with early gas leak detection capabilities [53]. In this case, the optimal PID tuning procedure described in this paper is responsible for the motion performances demonstrated by the APR-02 mobile robot [47].

### 2.2. BDCM with an Embedded Low-Cost Magnetic Encoder

**Figure 2:**
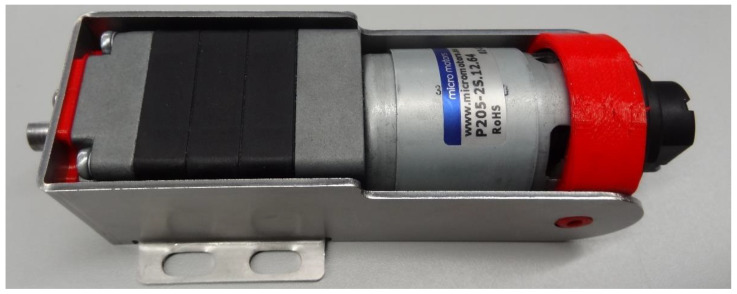
Image of the BDCM used in the APR-02 mobile robot, which includes a 64:1 planetary gearbox and a low-cost magnetic encoder attached to the motor shaft. The motor is supported by an aluminum support structure that has some (red) support elements made of flexible rubber in order to reduce the transmission of vibrations.

Figure 2 shows one of the three main driving motors used in the APR-02 omnidirectional mobile robot. This BDCM is the model P205-2S.12.64 (145.5 mm), manufactured by Micro Motors SRL (Verderio, Italy). This model is an industrial 12 VDC motor that includes on one side a gearbox with a ratio of 64:1 capable of delivering up to 6 Nm on the output shaft (diameter of 10 mm) and on the other side a low-cost magnetic quadrature encoder attached directly to the motor shaft.

The encoder has two hall-effect sensors and a six-pole magnetic ring: north–south–north–south–north–south. The hall-effect sensors include a temperature stability circuit, Schmitt trigger for signal stability, and open-drain MOSFET digital output. The sensors can operate with supply voltages between 3.5 V and 24 V and the output can sink up to 20 mA. The hall-effect sensors are placed at approximately a 90° phase offset and generate two output digital signals, A and B, providing a quadrature signal that encodes both the angular rotational velocity and direction of the angular rotation. Finally, a complete rotation of the motor generates three digital pulses in each encoder output as a consequence of the rotation of the six-pole magnetic ring: each north pole generates a high (or 1) digital output when over a hall-effect sensor and each south pole generates a low (or 0) digital output when over a hall-effect sensor.

### 2.3. Electronic Control Board Implementing the PID Controller

**Figure 3:**
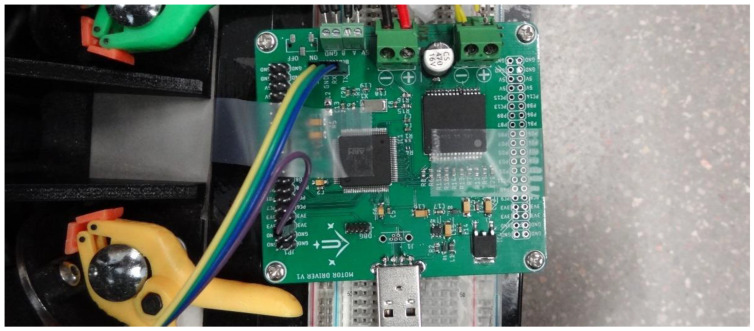
Electronic control board implementing the PID controller.

Figure 3 shows the electronic control board used in this paper to implement the PID controller of one BDCM, which is based on a microcontroller for signal and data processing and an H bridge for motor power control. The mobile robot APR-02 uses three of these BDCMs and its main electronic board is able to independently control these three motors.

The microcontroller used in this electronic board is the STM32F407VGT microcontroller designed by STMicroelectronics (Geneva, Switzerland), which includes a high-performance ARM Cortex-M4 32-bit RISC processor. This microcontroller is clocked at a frequency of 168 MHz, achieving a total of 210 DMIPS of processing power. The microcontroller includes standard peripherals for pulse-width modulation (PWM) generation, timers, and input capture interrupts used to measure the digital information provided by the encoders of the BDCM. Additionally, 196 kB of internal SRAM and 1 MB of flash memory are provided to store and run the user software. The effective power applied to the motor is adjusted by controlling the width of a 12 V digital pulse using a standard pulse-width modulation (PWM) strategy.

The H-bridge used in the electronic board is the VNH5019 from STMicroelectronics, a full-bridge motor driver designed for brushed DC motors capable of delivering up to 30 A and a maximum working voltage of 41 V. This H-bridge driver has two inputs that control whether the outputs are connected to the high- or low-voltage rails. Each input controls one of the outputs so that a logical 1 connects the output to the high-voltage rail (12 V in this paper) and a logical 0 connects it to the low-voltage rail (or ground). This control scheme prevents an accidental short circuit from the high-voltage rail to the low-voltage rail. An additional input is provided that enables or disables all switches of the H-bridge and is designed for the PWM signal that controls the power applied to the motor (allowing up to 20 kHz). Finally, the VNH5019 IC allows the monitoring of the current delivered to the load by generating a proportional output voltage. The frequency of the PWM signal used in this paper is 20 kHz and the voltage used to drive the DC motor is 12.0 V from a source capable of delivering up to 10.0 A.

### 2.4. PID Control Method with Anti-Wind-Up

**Figure 4:**
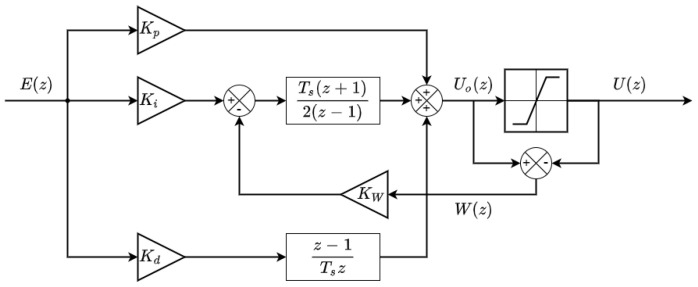
PID block diagram.

Figure 4 shows the block diagram of the discrete-time PID control method implemented in the APR-02 mobile robot to control the angular rotational velocity of the BDCM model P205-2S.12.64 (displayed in Figure 2). The PID control method implemented in the electronic board uses a discrete-time version of the classical PID proposal. The controller has been discretized using the Tustin method (or Trapezoidal method) for the integrator and the forward Euler method for the derivative part [9]. This implementation includes a classic anti-wind-up system to prevent the integrator accumulating a large error [9]; the conventional PID output control signal $u_{o}\left(k\right)$ is saturated in u(k) to limit the output control values to the physical limits of the control electronics and a negative feedback is introduced in the integrator part of the PID when the output control signal tends to overpass the saturated control signal. The $K_{w}$ parameter used in this implementation has been set to 39.025 according to the rule of thumb proposed by Dastjerdi et al. [54]:

(1) $$K_{w}=\frac{1}{\sqrt{\frac{K_{d}}{K_{i}}}}\;,$$

where $K_{d}$ and $K_{i}$ are the derivative and integrative gains of the reference controller described later in this paper. This anti-wind-up classical implementation introduces a nonlinear element that interferes with the normal behavior of a classical or theoretical PID controller, although its effects in the practical use of this controller are less harmful than the use of a nonsaturated integrator. There are also other anti-wind-up strategies available in the literature, such as that proposed by Huba et al. [55]. Finally, this PID controller is implemented as an isolated agent that is called at a regular interval defined by the sampling time $T_{s}$ by one of the internal timers of the microcontroller.

**Figure 5:**
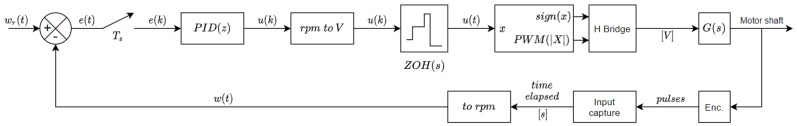
Representation of all steps and modules required to practically implement the PID controller of one motor of the APR-02 mobile robot.

Figure 5 represents all of the blocks implemented in the PID closed control loop, in which the PID part is executed periodically (every $T_{s}$) in the electronic control board. This control loop mixes discrete modules such as the PID(z) controller with the Laplace continuous model of the BDCM G(s) using a zero-order hold ZOH(s) in between. In this control loop, the target angular velocity $\omega_{r}\left(t\right)$ expressed in rpm units is compared with the current estimation of the angular rotational velocity of the motor $\omega_{r}\left(t\right)$, also in rpm units. Both angular velocities are expressed in rpm instead of rad/s or normalized units in order to simplify the practical application of the PID controller and the interpretation of the control task in the APR-02 mobile robot, which uses rpm in its path planning procedure [49]. The error signal e(t) expressed in rpm is sampled at $T_{s}$ intervals e(k) and applied to the discrete PID controller module, PID(z), which generates the discrete control signal u(k) used to control the motor, which is also expressed in rpm.

At this point it is interesting to note that the discrete control signal u(k) directly represents the rpm expected in the motor. For example, in a theoretical case with an ideal motor G(S)=1 and an ideal encoder that does not affect the measurement of the angular rotational speed of the motor, an example target velocity of 1500 rpm compared with a (perfect) angular velocity of 1500 rpm measured in the motor will generate an error signal *e*(*k*) = 0, but then the controller output u(k) needs to be 1500 rpm in order to make this ideal motor rotate at 1500 rpm. However, a real motor does not allow the application of an rpm value in the motor, so the controller output u(k) has to be converted into the voltage (or PWM percentage value) that will make the motor rotate to this 1500 rpm. In a real application with a BDCM, this conversion from rpm to V is linear and easy to determine experimentally. Finally, the voltage that must be applied to the mobile robot is applied in reality using a PWM signal that is directly deduced, considering that a duty cycle of 100% represents the application of 12 VDC in the motor (so 6 VDC requires a PWM of 50%).

In practice, the value of the control signal u(k) that must be applied as PWM to the H-Bridge is kept constant until the computation of the next loop by using the zero-order hold ZOH(s) module. Then, the H-Bridge applies the power to the motor and the rotation of the motor shaft generates digital pulses, whose length are measured using an input capture module of the microcontroller. At this point, it is interesting to note that counting the pulses of the encoder is not an option because counting the time elapsed between pulses generates detailed time information that is only practically limited by the resolution of the timer associated with the input capture function. The only drawback of this encoder measurement procedure is the low update rate provided when the motor rotates at very low angular velocities, but this problem is also present regardless of the encoder type used. Finally, the estimate of the angular rotational velocity of the motor, $\omega_{r}\left(t\right)$ in rpm can be compared with the target angular velocity $\omega_{r}\left(t\right)$ in the next control loop.

### 2.5. Error Funtion Used for PID Tuning Optimization

The optimization of the response of a PID controller requires a parametric comparison of different transitory results. The error function used in this paper is the normalized integral absolute error (NIAE) of the transient response, which is based on the work by Zhang et al. [56] and Zhenpeng et al. [57]. The NIAE applied in a step control signal applied at *t* = 0.0 s is defined as:

(2) $$\mathrm{NIAE}=\frac{\int_{0}^{T_{f}}\left|e\left(t\right)\right|dt}{\omega_{r}}\approx \overset{N}{\underset{K=0}{\sum }}\left|1-\frac{\omega \left(K\cdot T_{S}\right)}{\omega_{r}}\right|\cdot T_{S}$$

where $\omega_{r}$ is the amplitude of the step control signal used in the experiment (in this paper this target value is an angular rotational velocity of the motor expressed in rpm units), ω(t) is the instantaneous value of the angular rotational velocity of the motor provided by the magnetic quadrature encoder, N is the total amount of samples considered (that must be the same in all the measurements assessed), and $T_{S}$ is the sampling period or sampling rate of the controller. The computation of the NIAE aims to provide a numerical value that quantifies the absolute cumulative value of the step overshoot and undershoot responses generated by a specific PID controller configuration; this normalized value allows the comparison of the responses generated by the different target control values.

## 3. Practical Motor Modeling and Control

This section describes all of the practical steps used to optimally control the motors of the APR-02 mobile robot.

### 3.1. Optimal Measurement of the Angular Rotational Velocity Using a Magnetic Encoder

**Figure 6:**
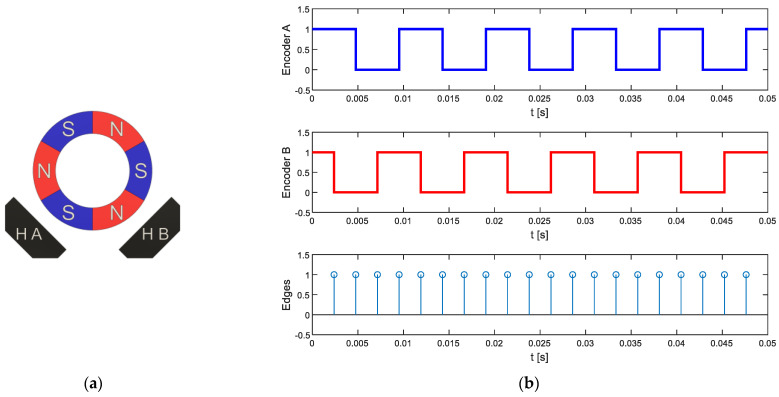
(**a**) Representation of the six-pole magnet of the encoder that is attached to the motor shaft and the two fixed hall-effect sensors, HA and HB, placed at a 90° phase offset. (**b**) Logical quadrature output signals generated by the rotation of the encoder and the edges detected by the microcontroller using the input capture module.

As previously described, the low-cost magnetic quadrature encoder originally embedded in the low-cost motorizations used in the APR-02 mobile robot generates three pulses per motor revolution in two output channels with a phase offset of approximately 90° between channels. Figure 6a shows a representation of the six-pole magnetic ring and the two hall-effect sensors and Figure 6b shows the typical quadrature signal generated by the encoder that simultaneously provides information for the angular rotational velocity and the direction of rotation of the motor.

The procedure used to deduce the angular rotational velocity of the motor or the wheel is based on the measurement of the time elapsed between the rising and falling edges of both quadrature output signals by using input capture interrupts in the microcontroller (Figure 6b). This time-elapsed measurement is used to directly estimate the instantaneous angular rotational velocity of the shaft of the motor. This measurement strategy produces a sequence of twelve time-elapse measurements,$\;\Delta t_{k=1\ldots 12}$, per motor revolution with a resolution that is only limited by the frequency of the timer used, which in this paper is 84 MHz. Equation (3) describes the expression used to estimate the angular rotational velocity of the wheel attached to the motor by taking into consideration that the magnetic encoder provides 12 rising and falling edges per revolution and that the gearbox has a reduction ration of 64:

(3) $$\omega_{\mathrm{WHEEL}}\left[\mathrm{RPM}\right]=\frac{1}{64\cdot 12\;\left[\frac{edges}{rev}\right]\cdot \Delta t_{k}\;\left[s\right]}\cdot \frac{60\left[s\right]}{1\left[\min\right]}$$

**Figure 7:**
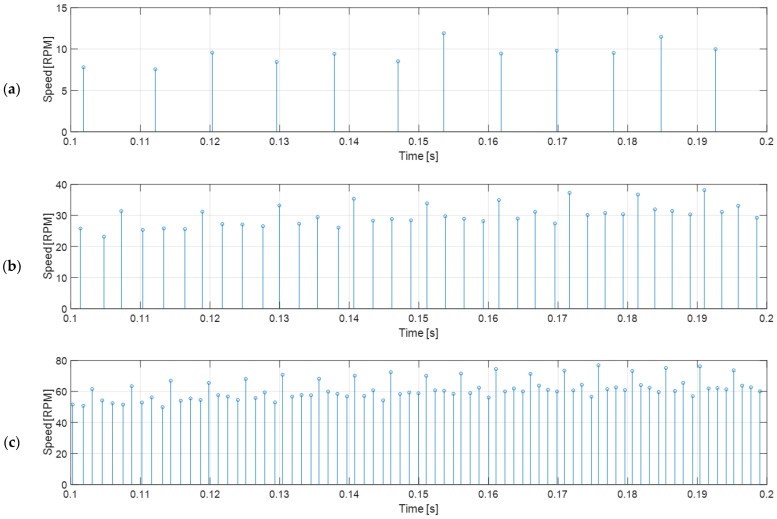
Open-loop wheel speed measurement deduced from the raw time-elapsed edge measurements gathered from the magnetic encoder of the BDCM for the different PWM duty cycles applied: (**a**) 20% or low-speed example; (**b**) 50% or medium-speed example; (**c**) 100% or full-speed example.

This measurement procedure provides an accurate update of the instantaneous angular rotational velocity of the wheel of the mobile robot whenever an edge is received from the encoder. The drawback of using low-cost magnetic encoders is the limited number of samples per motor axis rotation, although this strategy based on the time elapsed provides accurate measurements that cannot been obtained in the case of counting the rotations of the wheels [58]. This effect can be observed in Figure 7, which shows the estimation of the measurement speed of the wheel as a result of different PWM duty cycles (DC voltage or power) applied to the analyzed BDCM, at 20% (duty cycle equivalent to applying 1.2 V), 50% (equivalent to 6.0 V), and 100% (equivalent to 12.0 V); the respective wheel speeds measured are approximately 8 rpm, 30 rpm, and 60 rpm. Figure 7 shows that the throughput of information when the motor rotates at 30 rpm is approximately four times higher that when the motor rotates at 8 rpm, and when the motor rotates at 60 rpm it is approximately 8 times higher. This update rate effect will make the control of the motor at low angular rotational velocities difficult.

**Figure 8:**
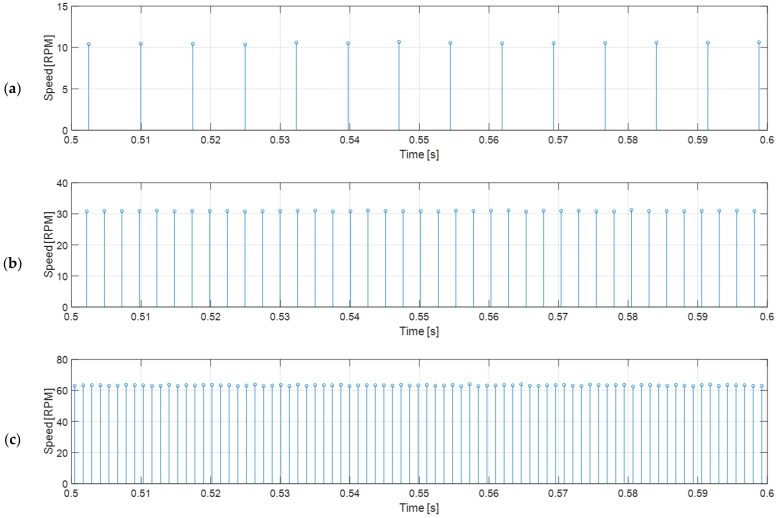
Corrected open-loop wheel speed measurement deduced from the raw time-elapsed edge measurements gathered from the magnetic encoder of the BDCM for the different PWM duty cycles applied and the correction coefficients displayed in Table 1: (**a**) 20% PWM case; (**b**) 50% PWM case; (**c**) 100% PWM case.

Figure 7 shows that the measured angular rotational velocity oscillates in a repeating pattern with a period of exactly 12 samples. This characteristic effect produced in low-cost magnetic encoders was firstly described and corrected by Palacin et al. [44], and is mainly caused by misalignments in the exact location of the magnetic fields in the magnet and in the exact location of the hall-effect sensors. This deterministic error can be corrected empirically by computing the average speed and the error coefficients that must be applied to each encoder reading in order to obtain the average value. The calibrated correction coefficients computed from the encoder readings shown in Figure 7b (BDCM placed in a no-load steady state with a PWM duty cycle of 50% and 12 V of source voltage) are listed in Table 1, and the applications of these correction coefficients to new readings are displayed in Figure 8. As can be seen comparing Figure 7 and Figure 8, the corrected angular rotational velocity readings gathered from the low-cost magnetic encoder are remarkably stable in open-loop measurements; therefore, this corrected velocity information will provide much more reliable feedback for the controller discussed later in this paper. Finally, as described in [44], the calibration of the correction coefficients of the low-cost magnetic encoder must be done only once during the manufacturing stage and stored in the microcontroller that must read the encoder and control the BDCM. The correct application of these calibration correction coefficients in each BDCM used in a mobile robot only requires an initial synchronization after a power-up.

### 3.2. Steady-State Motor Characterization

**Figure 9:**
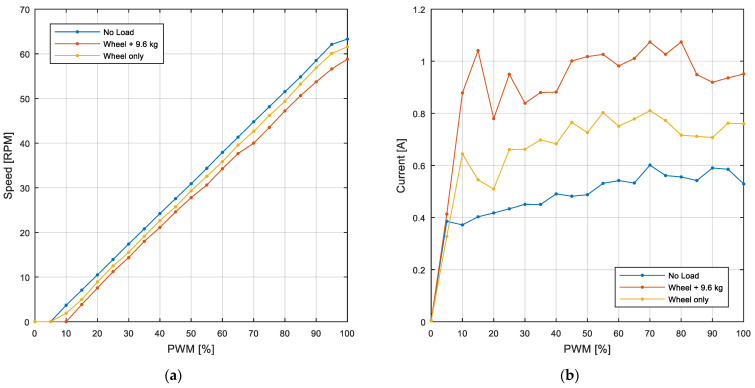
(**a**) PWM and RPM relationship in different load scenarios. (**b**) Motor current consumption depending on the applied PWM duty cycle in different load scenarios.

The example motor analyzed in this paper was experimentally evaluated with different loads and with different duty cycles in order to obtain a calibration curve to convert the PWM duty cycle to steady-state rpm (or vice versa). The loads analyzed include the motor without any load (free shaft), the motor with only an omnidirectional wheel attached in contact with the floor (3.2 kg), and the motor with the same wheel supporting the 9.6 kg that has to be supported in the APR-02 mobile robot. Figure 9a provides the steady-state relationship between the angular rotational speed of the output axis of the motor (axis of the wheel) and the open-loop PWM duty cycle applied, which is very linear in the PWM range from 10% to 95%. Additionally, Figure 9b provides the steady-state motor current measured in each experiment performed in Figure 9a.

Equation (4) shows the linear regression approximation required to estimate the PWM duty cycle (on a 0 to 100% scale) required to generate a specific angular rotational speed of the wheel (expressed in rpm) that is used in the “*rpm to V*” module of the PID controller of the motor (see Figure 5):

(4) $$\mathrm{PWM}\left[\%\right]=1.5667\cdot \left(\omega_{\mathrm{WHEEL}}\left[\mathrm{RPM}\right]+4.2229\right)$$

### 3.3. Open-Loop Motor Response Evaluation

**Figure 10:**
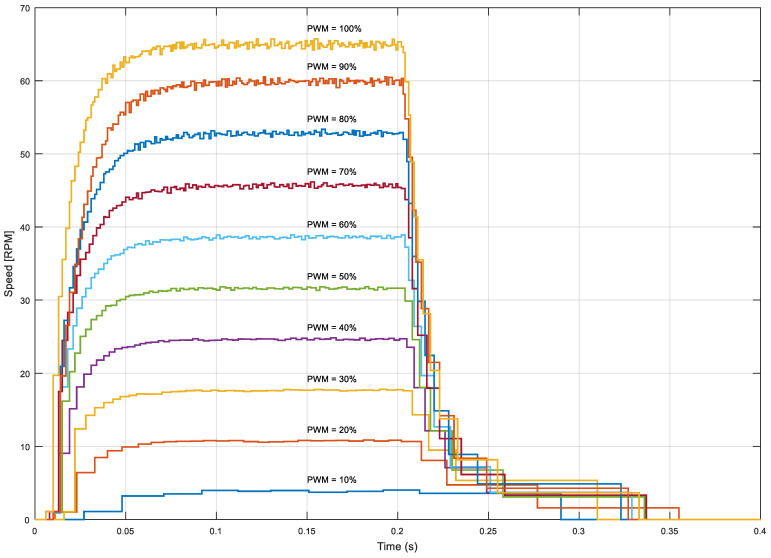
Motor step response for different PWM cycles.

This section is provided in order to shows the open-loop dynamic evolution of the angular rotational velocity of the omnidirectional wheel extracted from the information gathered from the low-cost magnetic encoder (the shown speed represents the output angular rotational speed of the wheel; to obtain the information at the motor shaft, just multiply the speed by 64). Figure 10 shows the open-loop dynamic evolution of the angular rotational velocity when applying a step from 0 to M% duty cycles with PWM at *t* = 0.0 s and a step from M to 0% at *t* = 0.2 s; the values of M are 10, 20, 30, 40, 50, 60, 70, 80, 90, and 100%. As described before, the number of speed samples provided by the encoder attached to the motor depends on the rotational angular speed of the motor as a side effect of the method used for the angular rotational velocity calculation. This effect reduces the amount of information gathered at low angular velocities (such as the duty cycle PWM = 10%) and when the motor is starting to rotate. Figure 10 shows that the cases in which a high PWM (such as 90 or 100%) is applied to the BDCM offer much more motor speed information than the cases with a low PWM (such as 10 or 20%), so the control of the motor will be easier at high speeds rather than at low speeds.

**Figure 11:**
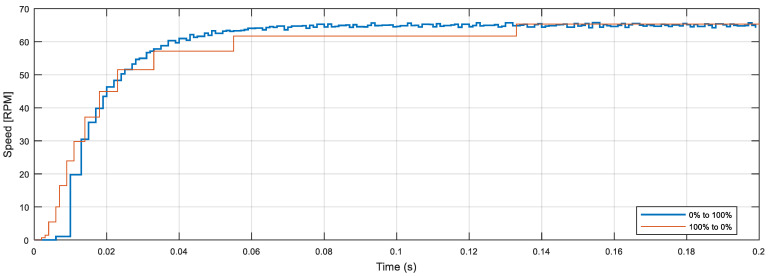
Acceleration curve and inverted deceleration curve moved to *t* = 0.0 s.

Finally, Figure 11 shows the details of the transitory result obtained in the case of applying a duty cycle PWM step from 0 to 100% at *t* = 0.0 s (blue line), in which the motor is accelerating, and also shows the deceleration obtained when applying a PWM step from 100 to 0%, obtained at *t* = 0.2 s (brown line), but in this figure it has been inverted (maximum speed minus instantaneous speed) and moved to *t* = 0.0 s in order to compare both the acceleration and deceleration curves. In a theoretical case, these two curves must be identical, so it should be expected that the combination of both transitory curves will provide a more detailed description of the open-loop response of the motor by overcoming the encoder’s limitations, for example using the information from the deceleration from 0 to 0.02 s and the information from the acceleration after 0.02 s. The specific practical application of the combined acceleration and deceleration curves will be analyzed in future works.

### 3.4. Motor Modeling

A theoretical model for the motor was created using the System Identification Toolbox (SIT) in Matlab^®^ software. This software program takes a dataset of measured input and output values from the studied plant and its sampling period to estimate the parameters of a given model structure that better fits the measured data. According to the Matlab^®^ documentation [59], the initial parameter values are defined using the simplified refined instrument variable method defined by Young et al. [60] and then adjusted using a nonlinear least-squares search method.

**Figure 12:**
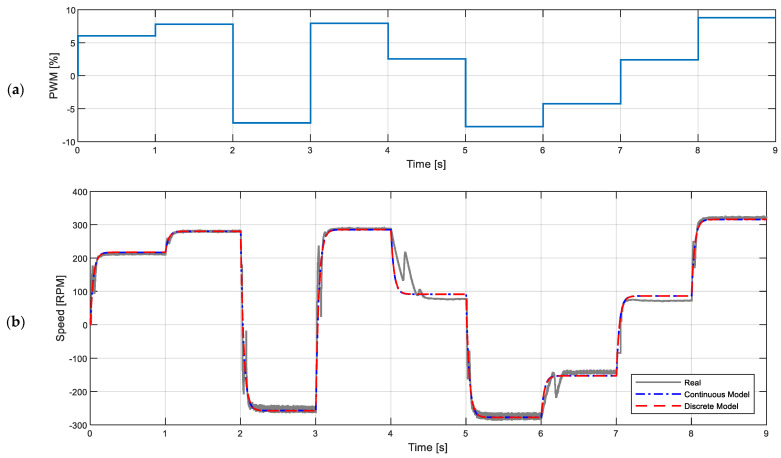
Calibration data required for model calculation: (**a**) PWM duty cycle sequence applied to the motor; (**b**) measured angular rotational velocity of the output shaft (gray line) and generated by the continuous-time (blue dotted line) and discrete-time (red dotted) models found by the SIT Toolbox.

The application of this motor modeling toolbox will be tailored to provide a transfer function model with two poles, no zeroes, and no delay, which is the standard function used in brushed DC motors. The calibration data used for this paper are composed with a sequence of 9 different PWM duty cycles applied to the motor during one second and the recording of the angular rotational velocity of the motor at a fixed sampling rate of 1 kHz. Figure 12a shows one sequence example at 50, 65, −60, 66, 35, −64, 35, 20, and 73%, and Figure 12b shows the recorded angular evolution and response obtained with two-pole continuous and discrete models. The transfer functions of the motor model obtained in continuous time and in discrete time (sampling time of $T_{s}=1\;\mathrm{ms}$) are:

(5) $$G\left(s\right)=\frac{1,858,880}{s^{2}+2080s+51,762}\;\left[\frac{\mathrm{rad}/s}{V}\right],\;\;G\left(z\right)=\frac{1.587}{1-0.1121z^{-1}-0.8437z^{-2}}\left[\frac{\mathrm{rad}/s}{V}\right]$$

Both expressions are provided in rad/s units by default. In general, the continuous model has applications in off-line analyses and simulations, while the discrete model is more suited for onboard mobile robot applications, because it allows the estimation or prediction of the behavior of the motors.

### 3.5. Model Validation Example

**Figure 13:**
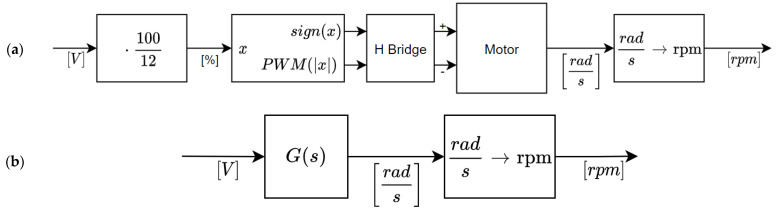
(**a**) Real motor setup. (**b**) Simulation of the motor model.

This section provides a specific validation example of the models obtained in the previous sections. Figure 13a symbolizes the real DC motor setup, where the voltage applied to the motor must be first converted to a PWM value and then applied to the H-bridge that controls the motor. Figure 13b symbolizes the blocks used to simulate the DC motor model in continuous time.

**Figure 14:**
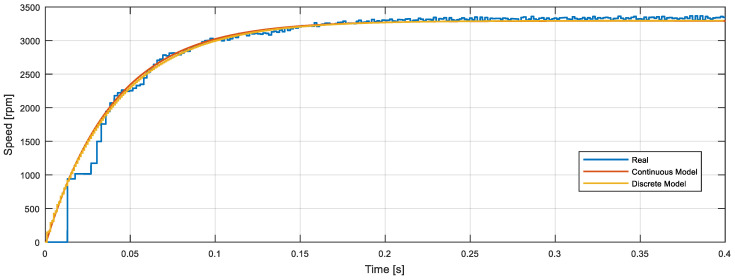
Open-loop motor step response comparison: real motor speed gathered from the encoder (blue line), continuous-time (red line) model simulation, and discrete-time (brown line) model simulation.

Figure 14 compares the step response (from 0 to 12 V at *t* = 0.0 s) obtained with the measurement of the real motor response and the simulation of the continuous and discrete models. Figure 14 shows the real information gathered from the magnetic encoder (blue line) and the typical theoretical evolution obtained from a function with two poles, no zeroes, and no delay.

### 3.6. Selection of the PID Sampling Period ($T_{s}$)

#### 3.6.1. The Sampling Theorem

The sampling frequency of the control loop is critical in the design of any digital controller system. The starting point for the selection of a sampling rate is the Shannon theorem or sampling theorem proposed by Shannon [61]. This theorem states that for a sampled signal to be properly reconstructed, it must have been sampled at a rate at least twice that of the maximum frequency component of the signal; that is, for a band limit frequency of $f_{b}$, the sampling frequency should be:

(6) $$f_{s}>2f_{b}$$

According to Santina et al. [62], in the case of a discrete-time control system, the closed-loop bandwidth can be used to determine a suitable sampling rate using the sampling theorem, suggesting a general rule of thumb through which the sampling rate should be in the range of:

(7) $$40f_{b}>f_{s}>10f_{b}$$

where $f_{s}$ is the sampling rate of the controller and $f_{b}$ is the closed-loop bandwidth of the system, which is an unknown parameter with expected values of around 10 Hz.

#### 3.6.2. Sampling Time Deduced form the Encoder Information

In this low-cost application, the real motor angular rotational velocity is estimated from the information gathered from a low-cost magnetic encoder. The nature of the speed calculation method generates speed samples at a variable sampling rate dependent on the instantaneous velocity of the motor. Therefore, at a certain point, increasing the sampling frequency of the controller will not increase the information gathered from the plant, since successive controller samples will obtain the same speed value from the magnetic encoder. Furthermore, at low motor velocities, the information from the encoder is updated at a slower rate, increasing the system delay and hindering its control. At any motor speed, the pulses per second generated by the encoder can be derived from Equation (3) using:

(8) $$f_{encoder}=\frac{1}{\Delta t_{k}}=\frac{\omega_{\mathrm{WHEEL}}\left[\mathrm{RPM}\right]\cdot 64\cdot 12}{60}\left[\mathrm{Hz}\right]$$

**Figure 15:**
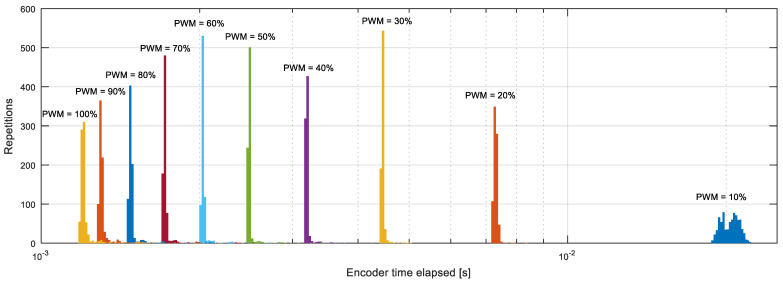
Histogram of the encoder’s time-elapsed ($\Delta t_{k}$) values obtained with different PWM duty cycles (cases represented in Figure 9a with no load), colored in order to differentiate the cases analyzed.

In the APR-02 mobile robot, the maximum angular rotational velocity of the wheels ($\omega_{\mathrm{WHEEL}}$) is around 64 RPM with a power supply of 12 V; consequently, the maximum encoder sampling rate is expected to be 819.2 Hz. As a validation of this value, Figure 15 shows the histogram of the time-elapsed values ($\Delta t_{k}$) measured from the encoder information at different PWM duty cycles represented previously in Figure 9a in the no load case. In this case, the minimum time elapsed measured from the encoder is 1.2 ms, so the initial proposed sampling period of the control system ($T_{s}$) is 1 ms. As stated before, it is pointless to reduce the sampling rate of the control system much above this encoder limit.

Finally, Table 2 summarizes the time elapsed measured from the edge signal provided by the quadrature encoder in the case of applying different PWM duty cycles to the motor. The update frequency of the rpm information gathered from the encoder is directly the inverse of the time elapsed. The value counted is the value of the 32-bit timer used to measure the time elapsed between edges by using the input capture module with a reference internal frequency of 84 MHz. This value is then used to estimate the angular rotational speed of the wheel and of the motor. Please note that the value counted has a very large resolution because it is higher than 100,000 (or 16 bits), even when the axis of the wheel rotates at its maximum velocity. Finally, the value of the counts per revolution is the estimated number of pulses that would be generated by an equivalent optical encoder in order to achieve the same resolution than the embedded low-cost quadrature encoder, which is a nonviable equivalent alternative. These high-resolution values acknowledge the efforts developed to correct the systematic encoder errors generated in the encoder.

### 3.7. Obtaining Baseline or Reference PID Parameters

**Figure 16:**
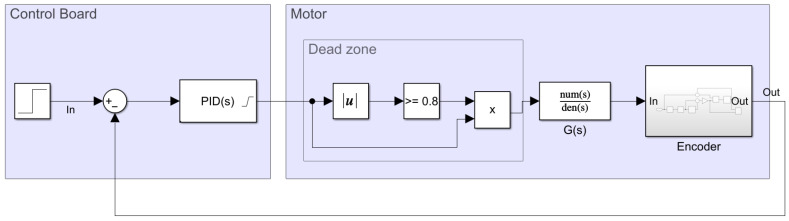
Simulink^®^ continuous control loop model used by the FRB PID tuner.

**Figure 17:**
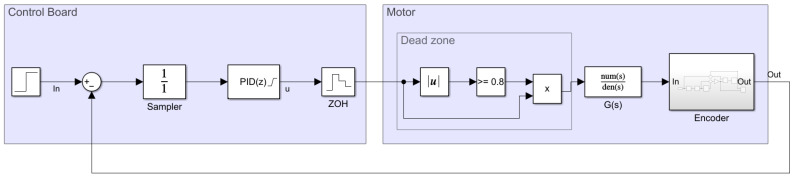
Simulink^®^ discrete control loop model used by the FRB PID tuner.

The behavior of a PID controller is defined by the value of its $K_{p}$, $K_{i}$, and $K_{d}$ parameters. The baseline or reference values of these controller parameters were obtained using the frequency-response-based (FRB) PID tuner integrated in the Simulink^®^ software [63]. This tool requires the creation of a control loop model in Simulink^®^ using the motor model previously described in this paper. The block description of this modeling is provided in Figure 16 and Figure 17 based on continuous and discrete PID controller implementations. The model of the motor contains various nonlinear elements that attempt to replicate the behavior of the real system. For improved reliability, the behavior of the encoder was also modeled in Simulink^®^ to be as close as possible to the real one by computing the rotation of the shaft of the motor, internally defining the quadrature encoder signal and finally providing a realistic time-elapsed sequence that is used to provide the same information as the real encoder.

The FRB PID tuner introduces a perturbation into the open-loop system and estimates the frequency response of the plant [63]. Then, the software uses these data to compute the PID parameters that better match the target bandwidth and phase margin specified by the user. The default target bandwidths proposed to reduce the step overshoot and phase margin are 40 rad/s (or 6.36 Hz) and 90°, respectively. The values of the PID parameters obtained with the FRB PID tuner are $K_{p}=1.5054$,$\;K_{i}=27.7177\;s^{-1}$, and $K_{d}=0.0182\;s$.

### 3.8. Basic Validation of the Baseline or Reference PID Parameters

**Figure 18:**
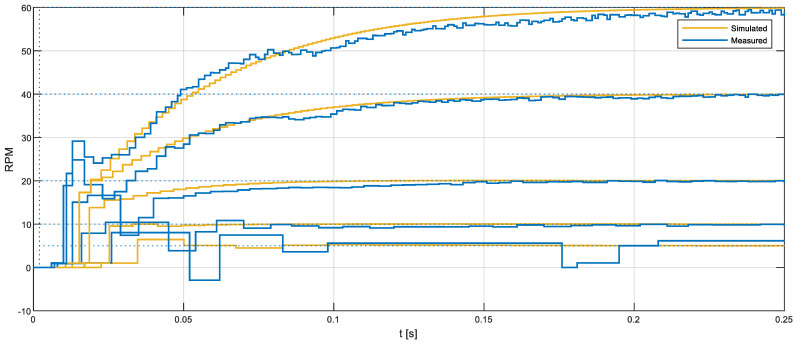
BDCM output in a closed-loop PID control: real evolution of the angular rotational velocity measured from the information gathered by the magnetic quadrature encoder (blue line) and simulated motor velocity (yellow line). Response to steps with target speeds of 5, 10, 20, 40, and 60 rpm.

Figure 18 shows a comparison between the real motor behavior (angular rotational velocity measured with the magnetic encoder) and the simulated behavior using a PID closed-loop control, with the baseline PID parameters obtained previously from the FRB PID tuner. Figure 18 shows the responses to the control steps with target speeds of 5, 10, 20, 40, and 60 rpm. On one hand, Figure 18 shows that the real implementation of the PID controller has difficulty when controlling the real DC motor at very low speeds (under 10 rpm). On the other hand, Figure 18 shows that the response of the PID in the cases of target speeds of 20, 40, and 60 rpm can be improved, because there is a peak and a valley undershoot during the acceleration of the mobile robot. This peak–valley response is not present in the simulation data and only appears in the real experimentation data provided by the magnetic encoder. The conclusion of this basic validation is that the baseline PID parameters obtained from the FRB PID tuner function applied to control this real DC motor that provides feedback using a low-cost magnetic encoder will require additional optimization in order to improve the response of the driving motors used in the APR-02 mobile robot, because a robotic application is a very demanding PID application that requires the minimum overshoot and undershoot in order to follow the planned trajectory ensuring the minimum possible structural error.

### 3.9. Validation of the Sampling Rate ($T_{s}$) of the PID Controller

**Figure 19:**
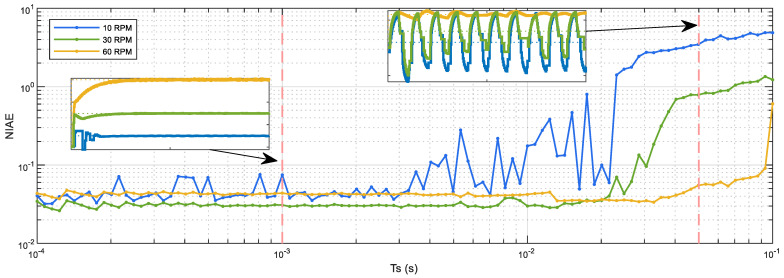
Evaluation of the NIAE values for different sampling periods ($T_{s}$) and different target speeds: 10 (blue line), 30 (green line), and 60 (yellow line) rpm.

This section proposes the empirical validation of the sampling rate ($T_{s}$) used in the implementation of the PID controller. The sampling rate used in the previous sections was 1 ms and was determined from the encoder data rate (see Section 3.6.2). Figure 19 shows the empirical relationship between the NIAE and the sampling rate used in the implementation of the PID controller. Figure 19 shows the experimental data obtained with the real motor in the case of step target speeds of 10, 30, and 60 rpm. During these experiments the PID controller was configured with the baseline or reference PID parameters and the sampling rate was changed between 0.1 ms and 0.1 s using logarithmic spacing. In general, Figure 19 shows that the NIAE is approximately constant between 0.1 ms and 3 ms, and from this value the NIAE corresponding to a target speed of 10 rpm starts to grow. The NIAE obtained with the medium target speed tested (30 rpm) starts to grow at approximately 20 ms, while the NIAE obtained with the highest target speed tested (60 rpm) starts to grow at approximately 40 ms. Figure 19 also presents the dynamic step response obtained in two sampling time cases, 1 ms and 45 ms, which are overlapped in the figure. In the case of using a sampling time of 1 ms, the angular rotational velocity regulated with the PID controller has some initial oscillations in the case of a target speed of 10 rpm and requires some time in order to reach the highest target speed (60 rpm). In the case of using a sampling time of 45 ms, the angular rotational velocity regulated with the PID controller oscillates with low and medium target speeds and shows some ripples in the case of the highest target speed. These results agree with the rule of thumb [62] that suggests the use of sampling rates of 10 or more times the system bandwidth. Therefore, according to the NIAE evolution displayed in Figure 19, any sampling rate between 0.1 ms and 3 ms will be valid, so the initial selection proposed in Section 3.6.2 ($T_{s}$ = 1 ms) is the sampling rate used in the practical implementation of the PID controller applied in the APR-02 mobile robot.

### 3.10. Optimization of the PID Parameters for Minimum Overshoot and Undershoot

This final section presents the method used to optimize the values of the PID parameters in order to minimize the overshoot and undershoot according to the computation of the NIAE parameter. This method requires a priori knowledge of a set of PID controller parameter values, which are used as reference points or as starting points in the design of robust PID controllers [64].

**Figure 20:**
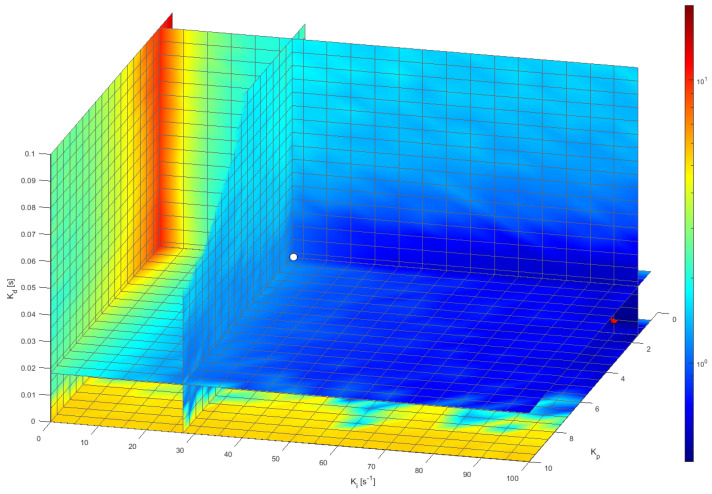
A 3D representation of the NIAE of the PID controller according the planes defined by the $K_{p}$, $K_{i}$ and $K_{d}$ parameters. The white point depicts the location of the baseline values proposed by the FRB PID Tuner procedure. The leftmost plane is for $K_{i}=0\;s^{-1}$ and the bottom plane is for $K_{d}=0\;s$.

**Figure 21:**
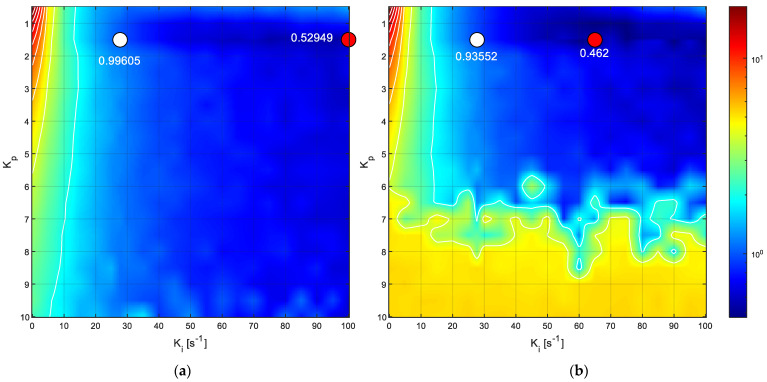
Details of the two horizontal planes defined in Figure 20. The white point depicts the location or projection of the NIAE baseline values obtained with the FRB PID tuner procedure while the red point depicts the location of the minimum NIAE in each plane. The values of the NIAE are also displayed for reference: (**a**) plane with $K_{d}=0.0182\;s$; (**b**) plane with $K_{d}=0\;s$.

Figure 20 depicts the representation of the NIAE computed for a step target speed of 30 rpm represented in the 3D space defined by a range of feasible PID controller parameter values established from the default $K_{p}$, $K_{i}$, and $K_{d}$ parameter values proposed by the FRB PID tuner procedure. The range displayed in Figure 20 is $K_{p}$ from 0.5 to 10 in steps of 0.5, $K_{i}$ from 0 s^−1^ to 100 s^−1^ in steps of 5 s^−1^, and $K_{d}$ from 0 s to 0.1 s in steps of 0.005 s. The mesh defined in this 3D space was completely explored by performing 2121 experimental measurements with the real motor, each one consisting of applying a step with a target speed of 30 rpm for 1 s and waiting 0.5 s until the next measurement (in order to be sure that the motor is completely stopped). The value of a target speed of 30 rpm was used in this optimization because it is the most used wheel speed in the APR-02 mobile robot [48]. In total, this exhaustive or brute-force search was completed in 3181.5 s (about 53 min). The color scale used in Figure 20 represents the maximum NIAE in red and the minimum in blue. As could be expected, Figure 20 shows that the maximum NIAE (red color, worst overshoot and undershoot) is generated when $K_{p}$ and $K_{i}$ have small values (using a PID without P and I control terms). The white point represented in Figure 20 depicts the location of the reference PID parameters obtained from the FRB PID tuner procedure. Three central planes are shown specifically in order to provide visual information of the evolution of the NIAE in the planes defined by the reference PID parameters, and Figure 21 shows the details of the most interesting 2D horizontal planes obtained. The color representation used in Figure 20 and Figure 21 was 2D interpolated in order to enhance the visual interpretation of the data in the planes.

Figure 21a shows the details of the horizontal plane for $K_{d}$ = 0.0182 s (the default value proposed by the FRB PID tuner procedure), where the white point depicts the location of the NIAE computed with the default PID parameters, while the red point depicts the location of the minimum NIAE computed in this plane. Similarly, Figure 21b shows the horizontal plane for $K_{d}$ = 0 s, where the red point depicts the location of the minimum NIAE found in this plane, while the white point is the approximate projection of the NIAE computed with the default PID parameters. As a result of this exhaustive 3D search, the minimum NIAE value obtained was 0.462, corresponding to parameter values $K_{p}=1.5054$,$\;K_{i}=65.0\;s^{-1}$, and $K_{d}=0.0\;s$. These values differed by 0%, 134.5%, and −100%, respectively, from the reference PID parameters obtained with the FRB PID tuner procedure ($K_{p}=1.5054$,$\;K_{i}=27.7177\;s^{-1}$, and $K_{d}=0.0182\;s$), which provided a baseline reference NIAE value of 0.996, which represents an improvement of 53.6%.

**Figure 22:**
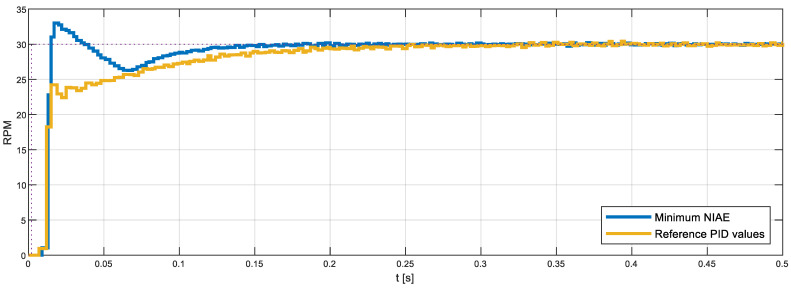
Step overshoot results measured in the real motor using the reference PID parameters obtained with the FRB PID tuner procedure (yellow line) and the best PID parameters, which minimize the NIAE. The target speed is 30 rpm.

Finally, Figure 22 compares the step responses generated with the reference PID parameters obtained with the FRB PID tuning procedure and the optimal PID parameters found, which minimize the NIAE (obtained empirically through an exhaustive search based on measuring the step transient in the real motor). Figure 22 shows that the optimal PID parameters generate a faster initial response in the DC motor while minimizing the undershoot. Please note that the overshoot and undershoot are computed as the cumulated absolute difference between the target velocity and the real motor velocity (see Equation (2)). The undershoot area generated with the reference PID parameters (NIAE = 0.99605) doubles the overshoot and undershoot areas obtained with the optimized PID parameters (NIAE = 0.462). This improvement was found to be similar in the angular rotational velocity range of the motor used in this paper. In the case of a mobile robot application using a PID control in the motors that drive the wheels, a minimum NIAE means a minimum difference between the target angular rotational velocity specified by the path planning algorithm and the expectation of a minimum trajectory divergence at the end of the motion.

## 4. Discussion and Conclusions

The use of a PID controller in a mobile robot is a very demanding application that requires the minimization of the overshoot and undershoot responses of the motor driving the wheels in order to accurately follow a planned trajectory. This paper has described the implementation of the PID controllers used in a human-sized omnidirectional mobile robot that uses three brushed direct current motors (BDCMs) to drive three omnidirectional wheels. The factors described and analyzed were the limitations of the angular velocity information gathered from the embedded low-cost magnetic quadrature encoder, the modeling of the brushed DC motor, the PID implementation, the anti-wind-up strategy used, the sampling time determination, the basic PID tuning procedure, and the optimization of the PID parameters for improved step overshoot and undershoot responses.

The electronic control board implementing the PID controller is based on an ARM microcontroller, which controls the BDCM through the duty cycle modulation of the PWM applied with an H-bridge. The electronic control board reads the velocity information from the magnetic quadrature encoder of the motor by using several input capture interrupts in order to measure the time elapsed between edges of the quadrature encoder output signal. In this case, a six-pole magnetic quadrature encoder is able to provide 12 instantaneous motor velocity updates per motor rotation, with effective resolutions of more than 16 bits at the maximum rotational speed of the motor (PWM duty cycle of 100%) and 21 bits (from the 32 bits available) at the minimum velocity (PWM duty cycle of 10%), values that cannot be reached with comparative optical encoders. Additionally, the angular rotational velocity provided by a low-cost magnetic encoder will have systematic errors originated by manufacturing inaccuracies. In this case, these systematic errors were corrected in order to take full advantage of the effective resolution provided by the input capture interrupts by applying a statistical methodology [44]. An analysis of the throughput of the time-elapsed information was used to define a strategy to select an appropriate sampling time in the PID controller.

The practical PID controller applied in an omnidirectional mobile robot to control three BDCMs is based on a discrete-time PID implementation using a classic anti-wind-up strategy to avoid the saturation of the integrator part of the controller. The brushed DC motor used in the mobile robot and in this paper has a linear relationship between the duty cycle of the PWM applied and the output angular rotational velocity. This linear relationship is used by the PID controller in order to compute the appropriate duty cycle to be applied to control the motor. The open-loop dynamic response of the BDCM was evaluated in a PWM duty cycle range of 10% to 100%, evidencing the difficulties of measuring the rotation of the motor at low angular velocities. The motor was modeled in order to obtain a typical two-pole model with no delay. The validation of this model in an open-loop control revealed the limitations of the measurements provided by the magnetic quadrature encoder when the motor rotates at very low angular velocities, limitations that are not usually considered in the continuous-time models of the motor used in the simulations.

The parameters of the PID controllers are primarily adjusted using the FRB PID tuner software. This tool is based on the generation of a random PWM duty cycle sequence and an analysis of the motor responses measured with the encoder. The reference PID parameters obtained with the FRB PID tuner software were $K_{p}=1.5054$,$\;K_{i}=27.7177\;s^{-1}$, and $K_{d}=0.0182\;s$. These reference PID parameters were validated experimentally by comparing the real and simulated responses of the motor. The experimental results showed that the real motor accelerated faster than expected at the start of the transitory phase, probably because the velocity information provided by the real encoder is zero rpm until the motor has rotated one-sixth of a turn (the measurement of the time elapsed requires the measurement of two edges), so the integer part of the PID controller tends to overaccelerate the motor in this starting stage until the reception of the first velocity information from the encoder. Obviously, this limitation is not present in the simulations because there are no encoder delay artifacts considered in the encoder models used in conventional simulations.

This paper has demonstrated that the effect of this low encoder throughput at low velocities causes a characteristic peak–valley behavior in the transient (see Figure 18) that has been considered as a possible cause of systematic trajectory errors in the mobile robot implementation. Therefore, the values of the PID parameters were optimized based on real experiments with the real feedback data provided by a low-cost embedded quadrature magnetic encoder by using a normalized integral of the absolute error (NIAE) as a feature function. The optimization of the PID parameters provided an optimal configuration that minimizes the overshoot and undershoot in the motor response with a NIAE reduction of 53.6% using $K_{p}=1.5054$,$\;K_{i}=65.0\;s^{-1},$ and $K_{d}=0.0\;s$. The application of this optimal PID tuning procedure in the APR-02 mobile robot is considered responsible for the motion performances achieved by this human-sized, three-wheeled omnidirectional mobile robot [47,48].

Although the exhaustive search method used to optimize the parameters of the PID controller used in this paper was implemented within a reasonable timeframe (performing 2121 validation experiments in 53 min), in a future study we will analyze the application of a Tabu search algorithm [25] as a procedure to optimize the search using the reference PID parameters as initialization search values and will dynamically extend this procedure to multiple setpoints. The advantage of the Tabu search algorithm is its ability to block the search directions that do not improve the transient response as a way to minimize the number of experimental measurements required in a practical optimization phase, a factor that is very important in the industrial manufacturing of commercial mobile robots using multiple sets of wheels.

Another planned study is the creation of a direct link between the PID controllers of the APR-02 mobile robot and a compact electronic nose (eNose) [65,66] in order to explore the field of reactive mobile robot olfaction (RMRO) in gas source localization applications [53].

## Figures and Tables

**Table 1:** Calibration of the low-cost magnetic encoder correction coefficients.

K1	K2	K3	K4	K5	K6	K7	K8	K9	K10	K11	K12
1.092197	0.886583	1.106404	0.941402	1.089113	0.892923	1.110171	0.934642	1.156358	0.839371	1.145867	0.949867

**Table 2:** Example encoder information obtained when applying a specific PWM to the BLDCM.

PWM	Wheel rpm	Time Elapsed [ms]	Update Frequency [Hz]	Value Counted	Counts per Revolution
10%	3.7	21.11	47.36	1,773,649	1,362,162,432
20%	10.8	7.23	138.24	607,639	466,666,752
30%	17.6	4.44	225.28	372,869	286,363,392
40%	24.5	3.19	313.60	267,857	205,714,176
50%	31.5	2.48	403.20	208,333	159,999,744
60%	38.6	2.02	494.08	170,013	130,569,984
70%	45.5	1.72	582.40	114,231	110,769,408
80%	52.8	1.48	675.84	124,290	95,454,720
90%	60.0	1.30	768.00	109,375	84,000,000
100%	64.4	1.21	824.32	101,902	78,260,736

## References

[1] Kuindersma S., Deits R., Fallon M., Valenzuela A., Dai H., Permenter F., Koolen T., Marion P., Tedrake R. (2016). Optimization-based locomotion planning, estimation, and control design for the atlas humanoid robot. Auton. Robot..

[2] Yeadon W.H., Yeadon A.W. (2001). Handbook of Small Electric Motors.

[3] Laughton M.A., Warne D.F. (2003). Electrical Engineer’s Reference Book.

[4] Zhou Y. (2013). Dc Motors, Speed Controls, Servo Systems: An Engineering Handbook.

[5] Aragon-Jurado D., Morgado-Estevez A., Perez-Peña F. (2018). Low-Cost Servomotor Driver for PFM Control. Sensors.

[6] Hijikata M., Miyagusuku R., Ozaki K. (2022). Wheel Arrangement of Four Omni Wheel Mobile Robot for Compactness. Appl. Sci..

[7] Yunardi R., Arifianto D., Bachtiar F., Intan Prananingrum J. (2021). Holonomic Implementation of Three Wheels Omnidirectional Mobile Robot using DC Motors. J. Robot. Control..

[8] Minorsky M. (1922). Directional Stability of Automatically Steered Bodies. Nav. Eng. J..

[9] Bennett S. (2001). The past of PID controllers. Annu. Rev. Control..

[10] Pallejà T., Saiz A., Tresanchez M., Moreno J., Ribó J., Clariá F. (2021). Didactic platform for DC motor speed and position control in Z-plane. ISA Trans..

[11] Grimholt C., Skogestad S. (2015). Improved Optimization-based Design of PID Controllers Using Exact Gradients. Comput. Aided Chem. Eng..

[12] Tabatabaei M., Barati-Boldaji R. (2014). Non-overshooting PD and PID controllers design. Automatika.

[13] Somefun O.A., Akingbade K., Dahunsi F. (2021). The dilemma of PID tuning. Annu. Rev. Control..

[14] Ziegler J.G., Nichols N.B. (1993). Optimum Settings for Automatic Controllers. J. Dyn. Sys. Meas. Control.

[15] Ang K.H., Chong G., Li Y. (2005). PID Control System Analysis, Design, and Technology. IEEE Trans. Control. Syst. Technol..

[16] Fruehauf P.S., Chien I., Lauritsen M.D. (1994). Simplified IMC-PID tuning rules. ISA Trans..

[17] Vilanova R. (2008). IMC based Robust PID design: Tuning guidelines and automatic tuning. J. Process Control..

[18] Ho W.K., Hang C.C., Cao L.S. (1995). Tuning of PID controllers based on gain and phase margin specifications. Automatica.

[19] Mikhalevich S.S., Baydali S.A., Manenti F. (2015). Development of a tunable method for PID controllers to achieve the desired phase margin. J. Process Control..

[20] Garrido J., Ruz M.L., Morilla F., Vázquez F. (2021). Iterative design of Centralized PID Controllers Based on Equivalent Loop Transfer Functions and Linear Programming. IEEE Access.

[21] Euzébio T.A., Da Silva M.T., Yamashita A.S. (2021). Decentralized PID Controller Tuning Based on Nonlinear Optimization to Minimize the Disturbance Effects in Coupled Loops. IEEE Access.

[22] Torga D.S., Da Silva M.T., Reis L.A., Euzébio T.A. (2022). Simultaneous tuning of cascade controllers based on nonlinear optimization. Trans. Inst. Meas. Control..

[23] Rachid A., Scali C. (1999). Control of overshoot in the step response of chemical processes. Comput. Chem. Eng..

[24] Lu Y.S., Cheng C.M., Cheng C.H. (2005). Non-overshooting PI control of variable-speed motor drives with sliding perturbation observers. Mechatronics.

[25] Bagis A. (2011). Tabu search algorithm based PID controller tuning for desired system specifications. J. Frankl. Inst..

[26] Mohsenizadeh N., Darbha S., Bhattacharyya S.P. Synthesis of PID controllers with guaranteed non-overshooting transient response. Proceedings of the IEEE Conference on Decision and Control and European Control Conference.

[27] Silva G.J., Datta A., Bhattacharyya S.P. (2002). New results on the synthesis of PID controllers. IEEE Trans. Autom. Control..

[28] Arciuolo T.F., Faezipour M. (2021). PID++: A Computationally Lightweight Humanoid Motion Control Algorithm. Sensors.

[29] Podlubny I. (1999). Fractional-Order Systems and PI^λ^D^μ^ –Controllers. IEEE Trans. Autom. Control..

[30] Efe M.Ö. (2011). Neural Network Assisted Computationally Simple PI^λ^D^μ^ Control of a Quadrotor UAV. IEEE Trans. Ind. Inform..

[31] Bruzzone L., Fanghella P. (2013). Fractional-Order Control of a Micrometric Linear Axis. J. Control. Sci. Eng..

[32] Birari A., Kharat A., Joshi P., Pakhare R., Datar U., Khotre V. Velocity control of omni drive robot using PID controller and dual feedback. Proceedings of the IEEE International Conference on Control, Measurement and Instrumentation (CMI).

[33] Meng J., Liu A., Yang Y., Wu Z., Xu Q. Two-Wheeled Robot Platform Based on PID Control. Proceedings of the International Conference on Information Science and Control Engineering (ICISCE).

[34] Suarin N.A.S., Pebrianti D., Ann N.Q., Bayuaji L., Syafrullah M., Riyanto I. (2019). Performance Evaluation of PID Controller Parameters Gain Optimization for Wheel Mobile Robot Based on Bat Algorithm and Particle Swarm Optimization. Lecture Notes in Electrical Engineering.

[35] Batayneh W., AbuRmaileh Y. (2020). Decentralized Motion Control for Omnidirectional Wheelchair Tracking Error Elimination Using PD-Fuzzy-P and GA-PID Controllers. Sensors.

[36] Megalingam R.K., Nagalla D., Nigam K., Gontu V., Allada P.K. PID based locomotion of multi-terrain robot using ROS platform. Proceedings of the International Conference on Inventive Systems and Control (ICISC).

[37] Wang J., Li M., Jiang W., Huang Y., Lin R. (2022). A Design of FPGA-Based Neural Network PID Controller for Motion Control System. Sensors.

[38] Borenstein J., Koren Y. (1987). Motion Control Analysis of a Mobile Robot. J. Dyn. Syst. Meas. Control.

[39] Borenstein J., Everett H.R., Feng L., Wehe D. (1997). Mobile Robot Positioning: Sensors and Techniques. J. Robot. Syst..

[40] Moreno J., Clotet E., Lupiañez R., Tresanchez M., Martínez D., Pallejà T., Casanovas J., Palacín J. (2016). Design, Implementation and Validation of the Three-Wheel Holonomic Motion System of the Assistant Personal Robot (APR). Sensors.

[41] Rubies E., Palacín J. (2020). Design and FDM/FFF Implementation of a Compact Omnidirectional Wheel for a Mobile Robot and Assessment of ABS and PLA Printing Materials. Robotics.

[42] Palacín J., Martínez D., Rubies E., Clotet E. (2021). Suboptimal Omnidirectional Wheel Design and Implementation. Sensors.

[43] Li Y., Ge S., Dai S., Zhao L., Yan X., Zheng Y., Shi Y. (2020). Kinematic Modeling of a Combined System of Multiple Mecanum-Wheeled Robots with Velocity Compensation. Sensors.

[44] Palacín J., Martínez D. (2021). Improving the Angular Velocity Measured with a Low-Cost Magnetic Rotary Encoder Attached to a Brushed DC Motor by Compensating Magnet and Hall-Effect Sensor Misalignments. Sensors.

[45] Qian J., Zi B., Wang D., Ma Y., Zhang D. (2017). The Design and Development of an Omni-Directional Mobile Robot Oriented to an Intelligent Manufacturing System. Sensors.

[46] Kao S.-T., Ho M.-T. (2021). Ball-Catching System Using Image Processing and an Omni-Directional Wheeled Mobile Robot. Sensors.

[47] Palacín J., Rubies E., Clotet E., Martínez D. (2021). Evaluation of the Path-Tracking Accuracy of a Three-Wheeled Omnidirectional Mobile Robot Designed as a Personal Assistant. Sensors.

[48] Palacín J., Rubies E., Clotet E. (2022). Systematic Odometry Error Evaluation and Correction in a Human-Sized Three-Wheeled Omnidirectional Mobile Robot Using Flower-Shaped Calibration Trajectories. Appl. Sci..

[49] Clotet E., Martínez D., Moreno J., Tresanchez M., Palacín J. (2016). Assistant Personal Robot (APR): Conception and Application of a Tele-Operated Assisted Living Robot. Sensors.

[50] Palacín J., Rubies E., Clotet E. (2022). The Assistant Personal Robot Project: From the APR-01 to the APR-02 Mobile Robot Prototypes. Designs.

[51] Palacín J., Clotet E., Martínez D., Moreno J., Tresanchez M. (2017). Automatic Supervision of Temperature, Humidity, and Luminance with an Assistant Personal Robot. J. Sens..

[52] Palacín J., Clotet E., Martínez D., Martínez D., Moreno J. (2019). Extending the Application of an Assistant Personal Robot as a Walk-Helper Tool. Robotics.

[53] Palacín J., Martínez D., Clotet E., Pallejà T., Burgués J., Fonollosa J., Pardo A., Marco S. (2019). Application of an Array of Metal-Oxide Semiconductor Gas Sensors in an Assistant Personal Robot for Early Gas Leak Detection. Sensors.

[54] Dastjerdi A.A., Saikumar N., HosseinNia S.H. (2018). Tuning guidelines for fractional order PID controllers: Rules of thumb. Mechatronics.

[55] Huba M., Chamraz S., Bistak P., Vrancic D. (2021). Making the PI and PID Controller Tuning Inspired by Ziegler and Nichols Precise and Reliable. Sensors.

[56] Zhang J., Zhuang J., Du H., Wang S. (2009). Self-organizing genetic algorithm based tuning of PID controllers. Inf. Sci..

[57] Zhenpeng Y., Jiandong W., Biao H., Zhenfu B. (2011). Performance assessment of PID control loops subject to setpoint changes. J. Process Control..

[58] Fiedeń M., Bałchanowski J. (2021). A Mobile Robot with Omnidirectional Tracks—Design and Experimental Research. Appl. Sci..

[59] Matlab Documentation: Tfest. https://es.mathworks.com/help/ident/ref/tfest.html?s_tid=srchtitle_tfest_1#btfb8zb-1.

[60] Young P., Jakeman A. (1980). Refined Instrumental Variable Methods of Recursive Time-Series Analysis Part III. Extensions. Int. J. Control..

[61] Shannon C.E. (1949). Communication in the Presence of Noise. Proc. IRE.

[62] Santina M.S., Stubberud A.R., Hostetter G.H., Levine W.S. (1995). Sample-Rate Selection. The Control Handbook.

[63] Matlab Documentation: Frequency-Response Based Tuning. https://es.mathworks.com/help/slcontrol/ug/frequency-response-based-tuning-basics.html.

[64] Silva G.J., Datta A., Bhattacharyya S.P. Robust control design using the PID controller. Proceedings of the IEEE Conference on Decision and Control.

[65] Palacín J., Rubies E., Clotet E. (2022). Classification of Three Volatiles Using a Single-Type eNose with Detailed Class-Map Visualization. Sensors.

[66] Palacín J., Clotet E., Rubies E. (2022). Assessing over Time Performance of an eNose Composed of 16 Single-Type MOX Gas Sensors Applied to Classify Two Volatiles. Chemosensors.

